# Population genetic considerations regarding the interpretation of within-patient SARS-CoV-2 polymorphism data

**DOI:** 10.1038/s41467-024-46261-4

**Published:** 2024-04-16

**Authors:** Vivak Soni, John W. Terbot, Jeffrey D. Jensen

**Affiliations:** 1 Center for Evolution & Medicine, Arizona State University, School of Life Sciences, Tempe, AZ USA; 2 Division of Biological Sciences, University of Montana, Missoula, MT USA

**Keywords:** Evolutionary genetics, Genetic variation, SARS-CoV-2

**arising from** H. Gu et al. *Nature Communications* 10.1038/s41467-023-37468-y (2023)

With the recent onset of the SARS-CoV-2 pandemic, there has been great interest in interpreting the within-patient evolutionary dynamics of this virus. Indeed, the accurate identification of genomic regions experiencing positive selection, and the quantification of these selective effects, is of crucial importance for both evolutionary as well as clinical interpretation. With this goal, the recently published Gu et al.^1^ work collected 2820 respiratory samples to investigate observed levels of within-patient synonymous relative to non-synonymous variation, and relied upon this comparison to assign genomic regions as evolving under purifying selection, neutrality, or positive selection. Specifically, they interpreted ${\pi }_{N}-{\pi }_{S}$ > 0 as being indicative of positive selection, ~0 as being indicative of neutrality, and <0 as being indicative of purifying selection (e.g., see Fig. 2 of Gu et al.). Using this criterion when performing sliding window analyses, the authors claimed that multiple genomic regions are experiencing positive selection. Crucially, the authors relied upon their selection inference derived from these $\pi$-based comparisons to support conclusions regarding infection dynamics in vaccinated vs. unvaccinated patients, a focal point of their publication.

There is a long history in the field of population genetics of comparing non-synonymous and synonymous divergence in this regard (i.e., d_*N*_/*d*_*S*_), as well as in jointly interpreting non-synonymous to synonymous divergence relative to polymorphism (e.g., as implemented in the McDonald-Kreitman test^2^, as well as numerous other related implementations; see refs. ^3,4^). In this framework, assuming that synonymous sites are evolving neutrally, the neutral divergence at these sites under genetic drift alone will be equal to the neutral mutation rate^5^, and thus non-synonymous divergence may be interpreted as being depressed by purifying selection or accelerated by positive selection relative to this synonymous/neutral standard.

However, this divergence-based interpretation does not correctly extend to a comparison of ${\pi }_{N}$ and ${\pi }_{S}$ as utilized by Gu et al. As one example, the effects of selection at linked sites (see review of ref. ^6^) renders this polymorphism-level interpretation problematic. Namely, even if mutations at synonymous sites are themselves neutral (and see ref. ^7^), their observed frequency in the population may be shaped by the episodic genetic hitchhiking effects associated with positive selection (i.e., selective sweeps^8^), and will be shaped by the constantly occurring genetic hitchhiking effects associated with purifying selection (i.e., background selection^9^). Importantly, these genetic hitchhiking effects will not impact divergence-based comparisons such as *d*_*N*_/*d*_*S*_ (^10^; though there are nonetheless important considerations, see refs. ^11,12^), but they will strongly impact polymorphism-based comparisons such as the ${\pi }_{N}-{\pi }_{S}$ of Gu et al.

For these reasons, one must account for the myriad of evolutionary forces shaping observed levels of within-patient nucleotide variation when performing population genomic inference of this sort^13,14^. In SARS-CoV-2 specifically, this evolutionary baseline model will necessarily include the underlying mutation and recombination rates, the history of population size change associated with infection, as well as the constant purging of deleterious mutations and the resulting effects on linked sites^15,16^. Only by accounting for these certain-to-be-operating evolutionary processes may one determine if episodic or hypothesized processes (such as positive or balancing section) need to be invoked to explain observed levels and patterns of variation^17–20^.

Thus, in order to investigate the claims of Gu et al., we simulated this SARS-CoV-2 baseline model in both the presence and absence of positive selection, in order to better interpret the behavior of ${\pi }_{N}-{\pi }_{S}$. As shown in Figs. 1 and 2, these simulations reveal multiple reasons to question their interpretations. Firstly, because of the small number of variable sites observed in the SARS-CoV-2 genome in any given patient sample, particularly after their filtering for SNPs segregating at greater than 2.5% frequency in a folded site frequency spectrum (i.e., resulting in a median of ~5 SNPs/sampled genome in the patient data), there is an extremely large variance associated with ${\pi }_{N}$ and ${\pi }_{S}$, which is only exacerbated by further reducing the scale of inference to specific genomic windows. For example, as shown in Fig. 1, in the complete absence of positive selection, it is naturally the case that purifying selection will on average reduce the frequencies of non-synonymous relative to synonymous variants (though the latter will be experiencing background selection effects); however, it is also the case that the variance is such that there is an appreciable probability of observing ${\pi }_{N}$ values that are larger than ${\pi }_{S}$ (i.e., their criteria for identifying positive selection), particularly on a sliding-window scale.

**Fig. 1:**
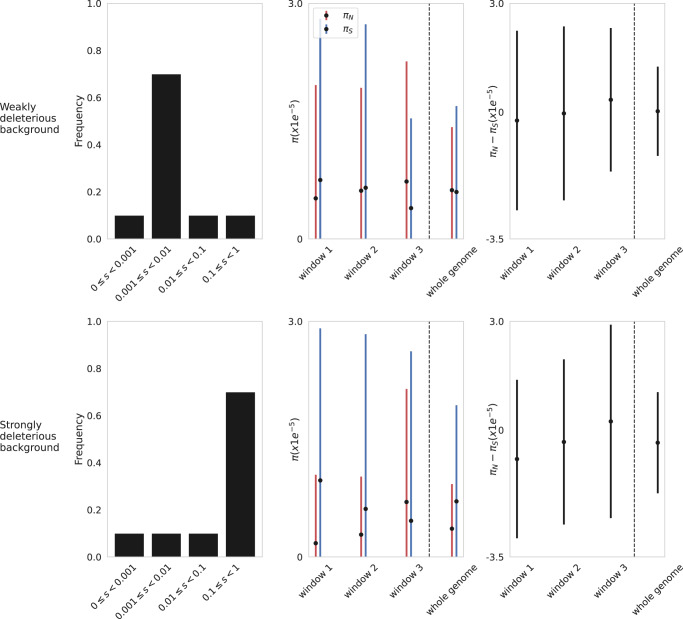
Per-site *π*_*N*_ and *π*_*S*_ values simulated under a model of primarily weakly deleterious mutations (top row), and a model of primarily strongly deleterious mutations (bottom row), occurring in the SARS-CoV-2 genome. The leftmost column provides the deleterious distribution of fitness effects (DFE) from which non-synonymous mutations were sampled under these two respective models; the middle column presents ${\pi }_{N}$ (red) and ${\pi }_{S}$ (blue) values for 10 kb non-overlapping windows of the genome, as well as the genome-wide values (30 kb); the rightmost column presents ${\pi }_{N}$ – ${\pi }_{S}$ values across the same genomic windows, and genome-wide. Point estimates represent mean values across 200 simulation replicates, with the standard deviation plotted as 68% confidence intervals. Simulations were performed using SLiM4.1^26^. Every third site of the genome was simulated as being strictly neutral (i.e., synonymous for the purpose of analysis), while all other sites were drawn from the respective DFE (i.e., non-synonymous for the purpose of analysis). Following the baseline model recommendations of refs. ^15,16^, the following parameterizations were utilized: infection bottleneck size = 1; recombination rate = 5.5e-5 events/site/cycle; mutation rate/site/replication = 2.135e-6; carrying capacity = 1e5. Simulations were run for 168 *N* generations (corresponding to an infection of 7 days), with 100 genomes sampled at the end-point. As per ref. ^1^, SNPs with an allele frequency less than 2.5% were masked when estimating $\pi$. Source data are provided as a Source Data file. All code for replicating these results is available on GitHub (https://github.com/vivaksoni/Gu_etal_2023_response).

**Fig. 2:**
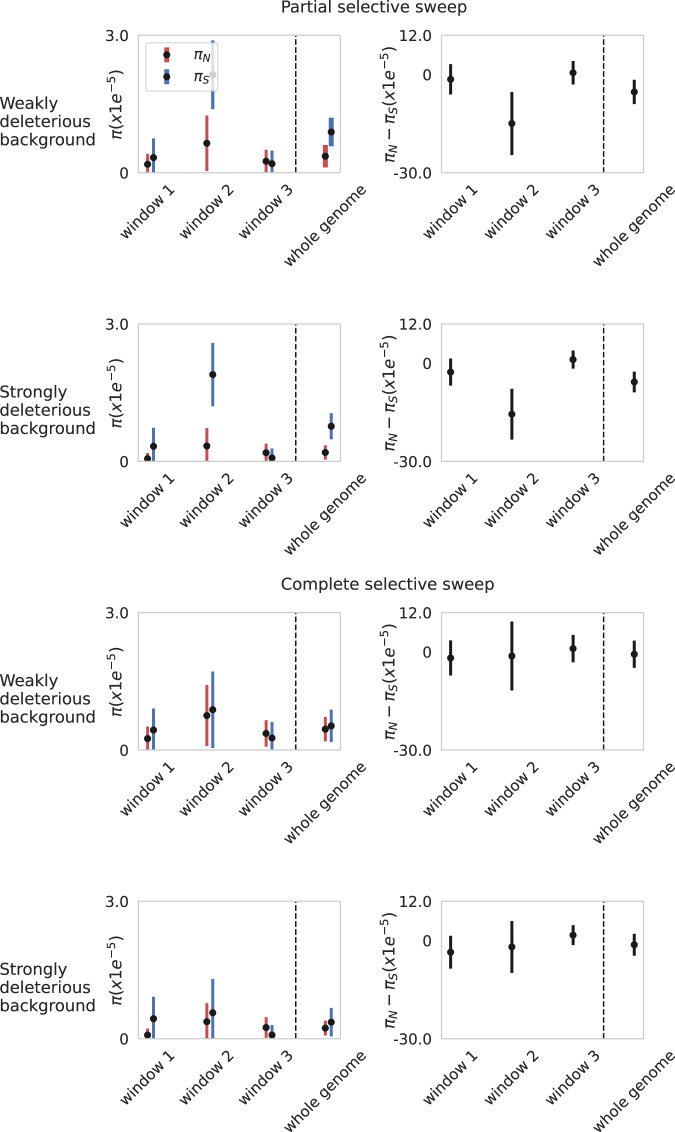
*π*_*N*_, *π*_*S*_, and *π*_*N*_-*π*_*S*_ values simulated under a model of a partial selective sweep (top panels) and a complete selective sweep (bottom panels), both on a weakly deleterious background as well as a strongly deleterious background (as given by the deleterious DFEs in Fig. 1). The top and bottom 2 × 2 plots present per-site ${\pi }_{N}$ (red) and ${\pi }_{S}$ (blue) values for 10 kb non-overlapping windows, as well as genome-wide (30 kb) values (left), and ${\pi }_{N}$ - ${\pi }_{S}$ values (right) across the same scales. Selective sweeps were modeled as a beneficial mutation with selection coefficient (*s*) = 10 introduced after 168*N* generations (7 days post-infection), in the middle of the simulated genome; sampling occurred when the beneficial mutation reached 50% frequency (partial sweep), and again at fixation (complete sweep). On average the beneficial mutation reached 50% frequency 14.8*N* generations and fixed 21.9*N* generations after introduction on the weakly deleterious background, and 15*N* generations and 22*N* generations, respectively on the strongly deleterious background. All other parameter details are in Fig. 1. Source data are provided as a Source Data file. All code for replicating these results is available on GitHub (https://github.com/vivaksoni/Gu_etal_2023_response).

Secondly, even in the presence of positive selection (Fig. 2), the implemented expectation of ${\pi }_{N}-{\pi }_{S}$ > 0 by Gu et al. would not successfully identify this evolutionary process. As shown for both a partial selective sweep (i.e., a beneficial mutation having reached 50% frequency in the patient population) and a complete selective sweep (i.e., a beneficial mutation having reached fixation in the patient population immediately prior to sampling), respectively, the expectation of ${\pi }_{N}-{\pi }_{S}$ remains negative. This observation partly owes to the fact that linked synonymous variants will be increased in frequency via genetic hitchhiking more readily than other linked non-synonymous variants which are likely deleterious; as such, synonymous variation in the hitchhiked region of the genome may be augmented more than non-synonymous variation. In addition, these models are similarly characterized by a large variance.

We additionally extended this model to consider recurrent beneficial mutations. Specifically, we evaluated scenarios in which 1% of new mutations are beneficial and in which 10% of new mutations are beneficial, occurring on the strongly or weakly deleterious DFE backgrounds given in Figs. 1 and 2, or occurring on the DFE background recently estimated for SARS-CoV-2 experimentally^21^. As shown in Supplementary Fig. 1, genomic windows were observed in all scenarios in which ${\pi }_{N}-{\pi }_{S}$ is both greater than and less than 0, and even genome-wide there is no significant differentiation in these distributions. It is worth emphasizing that while an extreme scenario in which 10% of all newly arising mutations are strongly beneficial and simultaneously segregating in the population may indeed elevate ${\pi }_{N}$ relative to ${\pi }_{S}$, even this unrealistic parameter space does not reliably produce this pattern. Furthermore, given that elevated ${\pi }_{N}$ may also be readily generated by models lacking positive selection entirely as shown, this $\pi$-based approach of Gu et al. remains inappropriate owing to issues of identifiability.

In summary, ${\pi }_{N}-{\pi }_{S}$ is not a reliable indicator of selective effects and dynamics. As shown in the specific case of SARS-CoV-2, the large variance associated with relatively few genomic SNPs renders the interpretation highly tenuous, leading to a situation in which values greater than 0 and less than 0 are both associated with appreciable probabilities in the presence of purifying selection alone. Furthermore, even with the addition of positive selection, the observation of ${\pi }_{N}$ > ${\pi }_{S}$ is unreliable owing partly to the effects of genetic hitchhiking. For these reasons, statistical inference procedures which directly account for multiple competing evolutionary processes (see refs. ^22,23^), and which utilize more sophisticated expectations associated with patterns of variation in the site frequency spectrum and linkage disequilibrium associated with positive selection (as reviewed by ref. ^24^, and see ref. ^25^), would be required to evaluate the claims of Gu et al.

## Reporting summary

Further information on research design is available in the Nature Portfolio Reporting Summary linked to this article.

### Supplementary information

Supplementary Information

Reporting Summary

### Source data

Source Data

## Data Availability

Datasets generated and/or analyzed during the current study are available in the paper. Source data are provided with this paper.

## Code availability

All scripts and data underlying the simulations, analyses, and Figures may be found at: https://github.com/vivaksoni/Gu_etal_2023_response.
