# Supplementary material for: Population genetic considerations regarding the interpretation of within-patient SARS-CoV-2 polymorphism data
Source: Nat Commun. 2024 Apr 16;15:3240. doi: 10.1038/s41467-024-46261-4 (PMC11021480; doi:10.1038/s41467-024-46261-4)
Supplement: Supplementary file 1 — Supplementary Information [file 41467_2024_46261_MOESM1_ESM.pdf]

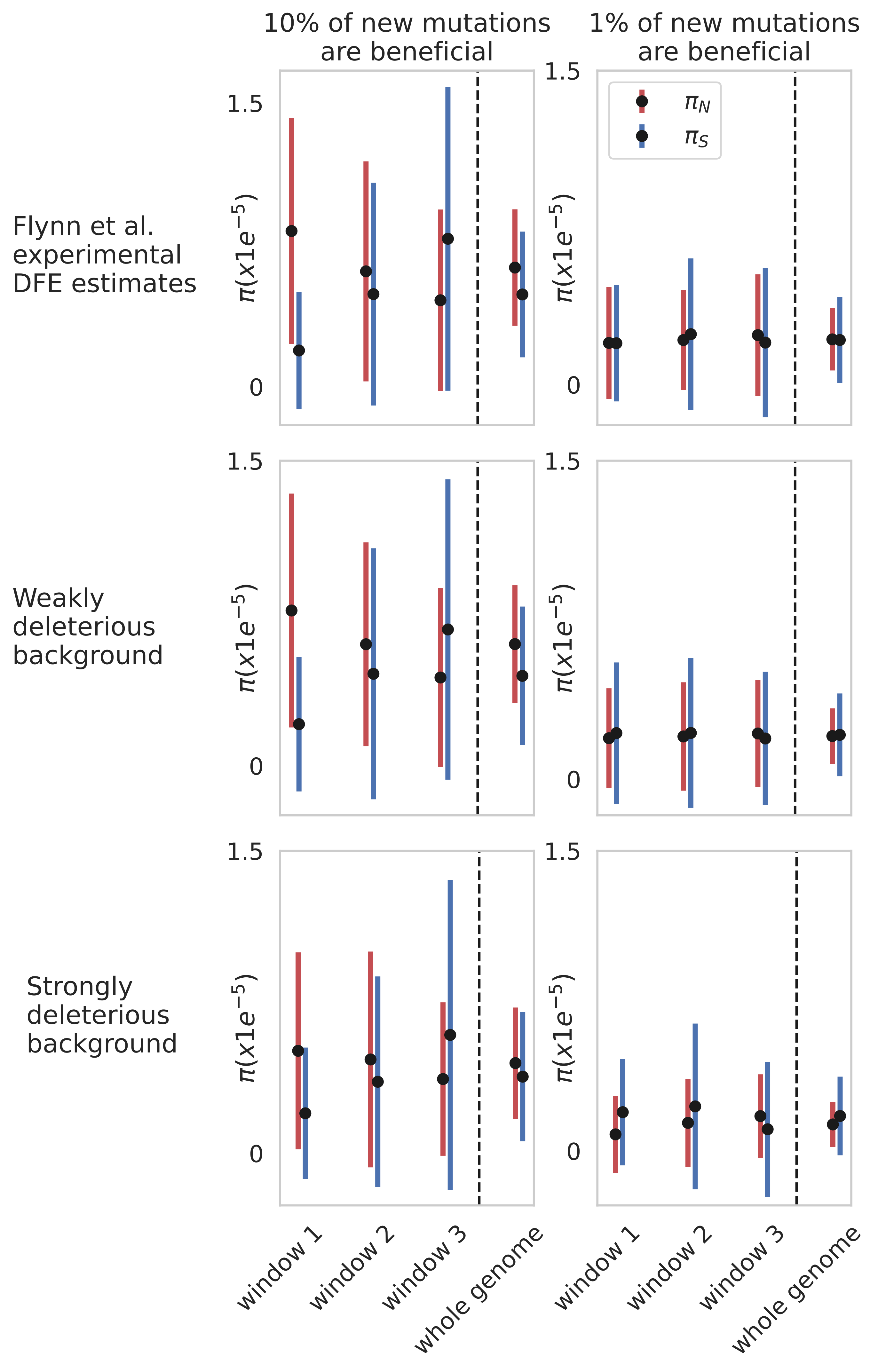

10% of new mutations are beneficial
1% of new mutations are beneficial
Flynn et al. experimental DFE estimates
Weakly deleterious background
Strongly deleterious background
$\pi(\mathrm{x1e}^{-5})$
1.5
0
π_N
π_S
window 1
window 2
window 3
whole genome

Supplementary Figure 1: Per-site π_N and π_S values simulated under recurrent beneficial mutation models, with 10% (left column) and 1% (right column) of mutations experiencing positive selection. These beneficial mutations are occurring on: the CoV-2 DFE experimentally inferred by Flynn *et al*. (2022) (top row), a weakly deleterious DFE as in the above Figures (middle row), and a strongly deleterious DFE as in the above Figures (bottom row). Plots present π_N (red) and π_S (blue) values for 10kb non-overlapping windows, as well as genome-wide (30kb) values. All other parameter details are as in Figure 1, except mutation rate/site/replication=2.135e-7 and carrying capacity=1e3, in order to achieve ~5 SNPs per sampled genome at >2.5% frequency (as in the empirical data) under these recurrent positive selection models (*i.e.,* the large input of beneficial mutations necessitates a reduced mutation rate relative to the above figures, in order to maintain levels of variation consistent with the published patient data). Source data are provided as a Source Data file. All code for replicating these results is available on GitHub (https://github.com/vivaksoni/Gu_etal_2023_response).
